# Highly interacting regions of the human genome are enriched with enhancers and bound by DNA repair proteins

**DOI:** 10.1038/s41598-019-40770-9

**Published:** 2019-03-14

**Authors:** Haitham Sobhy, Rajendra Kumar, Jacob Lewerentz, Ludvig Lizana, Per Stenberg

**Affiliations:** 1 Department of Molecular Biology, Umeå University, Umeå, Sweden; 2 Integrated Science Lab, Umeå University, Umeå, Sweden; 3 Department of Physics, Umeå University, Umeå, Sweden; 4 Division of CBRN Security and Defence, FOI–Swedish Defence Research Agency, Umeå, Sweden; 5 Department of Ecology and Environmental Science (EMG), Umeå University, Umeå, Sweden

## Abstract

In specific cases, chromatin clearly forms long-range loops that place distant regulatory elements in close proximity to transcription start sites, but we have limited understanding of many loops identified by Chromosome Conformation Capture (such as Hi-C) analyses. In efforts to elucidate their characteristics and functions, we have identified highly interacting regions (HIRs) using intra-chromosomal Hi-C datasets with a new computational method based on looking at the eigenvector that corresponds to the smallest eigenvalue (here unity). Analysis of these regions using ENCODE data shows that they are in general enriched in bound factors involved in DNA damage repair and have actively transcribed genes. However, both highly transcribed regions as well as transcriptionally inactive regions can form HIRs. The results also indicate that enhancers and super-enhancers in particular form long-range interactions within the same chromosome. The accumulation of DNA repair factors in most identified HIRs suggests that protection from DNA damage in these regions is essential for avoidance of detrimental rearrangements.

## Introduction

The chromatin in eukaryotic cells is not randomly organized, as various domains have been shown to occupy distinct ‘territories’ within the nucleus^1–4^. To decipher the chromatin architecture and three-dimensional (3D) organization within the nucleus, chromosome conformation capture techniques (such as 3C, 4C, 5C and Hi-C) have been developed^5–7^. In these techniques, chromatin segments in close spatial proximity are crosslinked, the crosslinked chromatin is digested and ligated, then the DNA is purified and sequenced. The chromatin segments identified as being in close physical proximity in this manner are considered as interacting loci. Finally, frequencies of interactions between pairs of loci are quantified. Visualization of chromosome conformation data as heat maps has revealed that the genome is partitioned into 3D compartments, *inter alia* topological associated domains (TADs) and A/B compartments^8–10^. Loci located within such domains tend to interact highly with each other and TADs’ boundaries are reportedly enriched in insulators and highly expressed genes^8,9,11–13^.

It has also been observed in other types of experiments that distant regions of the genome can interact^14^, and there are observations indicating that expressed genes tend to co-localize in the nucleus, forming so called transcription factories^5,15–18^. In *Drosophila* it has been shown that Polycomb repressed regions can also co-localize in foci^5,13,19–21^. In addition, DNA double strand breaks, were shown through fluorescence labelling to travel within the nucleus^22^ and breaks have also been shown to cluster together^23^.

Theoretically, there are obvious advantages in moving genomic regions that require similar factors into close three-dimensional proximity. However, bringing distant regions of the genome into proximity strongly raises risks of detrimental rearrangements if any DNA damage that occurs in such regions is not quickly repaired. Accordingly, there are indications that chromosomal rearrangements tend to occur in regions that are brought into three-dimensional proximity^24^.

Thus, the functional advantages of long-range interactions and associated 3-D conformations of DNA presumably outweigh the selective disadvantages of such risks. However, despite the efforts summarized above, knowledge of the nature and functions of many of the interactions is still rudimentary. Therefore, the aims of this study were to computationally define regions of the genome that form high numbers of long-range intra-chromosomal contacts using Hi-C data and investigate their properties using ENCODE data. For this purpose, we developed a new method to transform two-dimensional Hi-C contact maps into one-dimensional profiles. This method differs from TAD and A/B-finding techniques involving the construction of correlation matrices then finding clusters with Principal Component Analysis (PCA)^25^. Instead, our method involves direct use of Hi-C data (after a simple element-wise manipulation), and extraction of the eigenvector for the smallest eigenvalue (here, unity), where the values are proportional to the interactivity (or number of contacts) for a particular genomic region.

Using this method, we find that in line with previous observations some regions cluster by functions such as active transcription and Polycomb repression. In addition, we find that predicted enhancers and super-enhancers are potentially involved in long-range interactions and interestingly that most genomic regions with a high number of contacts are bound by DNA damage repair factors.

## Material and Methods

### Stationary distribution

To calculate the interactivity (or numbers of contacts) of genomic regions we consider each chromatin segment as a node in a network. The segment’s length is determined by the resolution of the Hi-C map, which thus also governs the numbers of nodes and links in the network. The links represent physical interactions. Since these interactions are not uniform across the genome, we assign weights to the links that are proportional to the frequencies that pairs of chromatin segments are physically close to each other in a cell population. We restrict the analysis to intra-chromosomal contacts (i.e. contacts within the same chromosome), since data on inter-chromosomal contacts in Hi-C are too sparse to include. The raw frequencies produced from a cell population could be directly taken as weights. However, contact frequency decays as a function of distance between chromatin loci, and contact frequencies are higher for neighbouring loci than for distant loci. Therefore, we derive weights from the raw Hi-C maps by subtracting each contact frequency with expected contact frequency, defined as the median contact frequency at each particular distance (Supplementary Information SI-1, 2 and Fig. S1). In the study reported here, Hi-C maps for GM12878 human lymphoblastoid cells at two resolutions (100 and 5 kb) were downloaded from the GEO database^7^. Next, we transformed the raw maps to ‘*observed* – *expected*’ maps using the gcMapExplorer package^26^ (https://github.com/rjdkmr/gcMapExplorer).

From the ‘*observed* – *expected*’ map, we construct a transition probability matrix *W*, where every entry is the probability to jump from one node to any other node in the network. To calculate *W*, we divide every row in the ‘*observed* – *expected*’ map by its sum (shown as a network in Fig. S1 and Supplementary Information SI-2). Based on *W*, we then formulate a Markov model for how a particle randomly jumps between nodes in the network. Denoting ${P}_{1}(n),{P}_{2}(n),\mathrm{...},{P}_{N}(n)$ as the probability that the particle is in node 1,2, …*N* at time *n*, we calculate ${P}_{1}(n+1),{P}_{2}(n+1),\mathrm{...},{P}_{N}(n+1)$ as a simple one-step process.

$$p(n+1)=p(n)W,$$

where

$$p(n)=[{P}_{1}(n),{P}_{2}(n),\mathrm{...},{P}_{N}(n)]$$

From this, we are interested in the stationary probability distribution $p(n=\infty )={p}^{\infty }$, which we obtain from the equation ${p}^{\infty }={p}^{\infty }W$. This means that ${p}^{\infty }$ is the normalised eigenvector of *W* associated with the eigenvalue one. In this study, to calculate ${p}^{\infty }$ we used the Scipy Python library to eigendecompose the transition probability matrix *W*, and extracted the eigenvector with the largest eigenvalue (one in this case). The above method is implemented in the gcMapExplorer package^26^ (https://github.com/rjdkmr/gcMapExplorer), and the user can easily calculate the stationary distribution from a raw Hi-C map in a few steps.

When the raw Hi-C map was directly used to calculate the Transition Probability Matrix (TPM), the dynamic range in the resulting stationary probability distribution (SPD) was limited due to the inclusion of both expected neighbouring and short-range contacts as well as long-range contacts (Fig. S2). Observed/expected’ normalization did not improve the dynamic range of the resulting SPD. When ‘observed-expected’ normalization was used to calculate the TPM, the SPD had a very similar profile but larger dynamic range (Fig. S2), thus it was applied in further analyses.

### Chromatin-related datasets and correlation

We downloaded ChIP-seq data for 177 chromatin-related datasets (available for GM12878 cells) from the ENCODE project website (https://www.encodeproject.org/), last updated in 2016. The ENCODE dataset contains ChIP-seq data for 87 chromatin-bound proteins (listed in SI-3), as well as various histone modifications, CG methylation, DNA accessibility (DNase-seq) and nucleosome density (MNase-seq), hereafter ENCODE factors (177 in total). We used these data to calculate global correlations between the stationary distribution and ENCODE factors. The Spearman correlation between the average (for 100 kb windows) Hi-C stationary distribution values and average enrichment of these variables was calculated across chromosomes 1, 2, 6, 7, 8, 9, 10, 11, 20, 21, 22 and X (Supplementary Information SI-4).

### Defining the genomic regions with the highest numbers of contacts

The genomic regions with the highest numbers of contacts (Highly Interactive Regions, HIRs) were defined as regions consisting of five or more consecutive 5 kb bins (the original Hi-C map resolution^7^) with stationary distribution values exceeding the 90^th^ percentile for each chromosome (Fig. S3). In this manner we identified 787 HIRs across the genome with an average length of 31.2 kb (Fig. S4 and Supplementary Information SI-5, 6).

### Enrichment at HIRs

Before constructing boxplots or heat-maps and PCA, the ChIP-seq datasets were normalized to bring their enrichment values within a similar range, as follows. First, all values below the genomic average for each individual dataset were set to the genomic average value. Then each value was replaced by the corresponding percentile value, computed from the distribution of values for each dataset after downsampling the data (using averages) to 5 kb resolution. Following these procedures, a value of zero represents enrichment at or below the genomic average level (background levels), and a value of 100 represents the maximum enrichment observed in the genome. Datasets with >50% missing data or average enrichment after normalization below the second percentile at HIRs were excluded.

The Principal Component Analysis (PCA) was performed using SIMCA (Umetrics). Only the six components that was determined to be significant in the software was used to perform Ward clustering where six classes of HIRs, which we designated HIR1-HIR6 were defined. Clustering was also performed in SIMCA.

### Overlapping HIRs with other datasets

HIRs regions were overlapped with features drawn from the following sources (Supplementary Information SI 7–9). Reference genes and gene expression level (RNA-seq data) were downloaded from ENCODE and Ensembl database (http://www.ensembl.org), hg19, and was used to compute RPKM values for each gene using QuickNGS^27^. Non-coding RNAs (ncRNA) were drawn from RNA central (http://rnacentral.org/). Predicted typical and super-enhancers, TADs, and frequently interacting regions (FIREs) were obtained from published sources^7,28,29^. Sites of double-strand breaks (DSBs) induced by aphidicolin or neocarzinostatin (which respectively mimic DSBs caused by replication stress and radiation) were also obtained from a previous publication^30^. Genomic coordinates of features mapped to the hg18 reference genome in these datasets were converted to hg19 coordinates using liftOver.

We used TADs defined in the original publication from where we obtained the Hi-C data^7^. TADs where downloaded as a table with defined regions between a start and a stop position. If a HIR overlapped a start or stop position it was classified as overlapping a TAD border. If it fell entirely within a TAD, it was defined as being inside a TAD.

### Expected values

To calculate expected densities of genes, enhancers, and DNA breaks we calculated their expected numbers in regions the size of HIRs under the assumption that they are evenly distributed throughout the genome. Furthermore, HiC-SD_0–30_, HiC_30–50_ and HiC_50–80_ regions were calculated in the same manner, as HIRs within <30^th^, 30^th^-50^th^ and 50^th^–80th percentiles, respectively.

### Contact frequencies between different classes

We calculated a map of‘*observed*/*expected*’ contact frequencies from the raw Hi-C map for GM12878 cells using the gcMapExplorer package, extracted contact frequencies between loci either within the same HIR class or different classes (generated by the PCA and clustering) from the map and generated violin distribution plots. Additionally, for genome-wide comparison, we generated violin plots of contacts between each class and 10000 randomly selected loci (from the whole genome) that do not overlap that particular class. In these violin distribution plots, ‘*observed*/*expected*’ contact values for a pair of loci larger than one indicate higher than expected contact frequencies.

## Results

### Identification of highly interacting genomic sites within chromosomes

**Figure 1:**
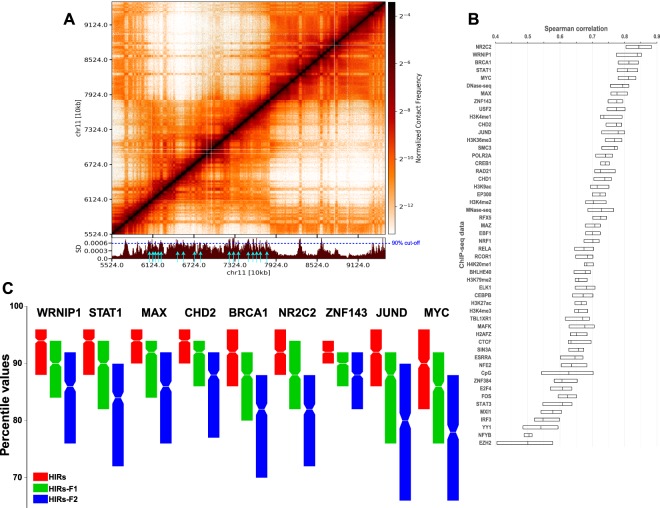
(**A**) A genome browser screenshot (gcMapExplorer^26^) of the Hi-C contact map for GM12878 cells at 10 kb resolution, the lower panel shows the stationary distribution (SD). Highly interacting regions (HIRs) are indicated by light blue arrows, Table S1. (**B**) Spearman rank correlations between Hi-C stationary distribution and ENCODE factors at 100 kb resolution. For the most strongly correlated factors, see Supplementary Information SI-4. (**C**) The boxplot shows enrichment of factors in the HIRs compared to the two flanking regions. The ChIP-seq values were normalized to the corresponding percentile values (for additional factors see Fig. S7). The factors are significantly more enriched in HIRs than in flanking regions (student t-test, all p-values < 0.005).

The contact probability matrix from a Hi-C experiment gives the pairwise contact probabilities between different genomic positions. In this study our aim was to understand which genomic regions are forming the highest number of contacts with other regions and therefore might constitute structural interaction hubs and/or represent regions that need to be quickly found by e.g. regulatory factors searching for their targets along chromosomes. To identify these highly interacting regions, we have adapted and applied the Markov stochastic process on Hi-C data. Here we interpret the Hi-C map as a network with weighted links that reflect the probability that two segments of the chromatin co-localises in three-dimensional space. Considering diffusion in the contact network, we asked, what is the probability that some factor searching along the chromosome is in a specific node, and which nodes have the highest probability and hence are the most accessible? For this purpose, we calculated the stationary probability distribution for each chromosome (see *Material and Methods*) using Hi-C data for the human lymphoblastoid cell line GM12878^7^. Regions with high stationary distribution and thus high numbers of contacts (within the chromosomes) are distributed across the chromosomes (Fig. S5) and seem to localize in a subset of TADs (Fig. 1A, Supplementary Information SI-2). Since inter-chromosomal interactions are poorly represented in the Hi-C data we restricted all analyses in this study to intra-chromosomal interactions.

### Highly interacting regions are enriched in DNA repair factors

To identify proteins or other regulatory factors that tend to accumulate at highly interacting sites of the genome we correlated the stationary distribution with maps of 177 factors in GM12878 cells according to 177 ENCODE datasets (see Supplementary Information SI-3 for a complete list). For this, we calculated Spearman rank correlation coefficients (R) between the stationary distribution and mapped factors at 100 kb resolution (SI-3, 4), and found strong correlations (R ≥ 0.5 and p < 0.05) with 54 (Fig. 1B). The most strongly correlated proteins (including NR2C2, WRNIP1, BRCA1, STAT1 and MYC) are all involved in transcription and repair of DNA damage and breaks (GO-analysis in Fig. S6). Cells lacking BRCA1 are very sensitive to DNA-damaging agents and develop chromosomal aberrations^31^. BRCA1 and WRNIP1 are allocated at stalled replication forks to protect them from degradation and promote fork restart after replication stress^32–34^. MYC is involved in radiotolerance and can activate the Ataxia telangiectasia mutated (ATM)-dependent DNA damage checkpoint response^35,36^. DNA damage leads to activation of interferon-stimulated genes (interferon signalling), including STAT transcription factors^37^. NR2C2 is recruited by poly (ADP-ribose) polymerase 1 (PARP1) at damaged loci^38^.

Other highly correlated datasets, including H2AFZ, SMC3, CTCF, DNase, MNase, and a number of histone marks, are known to have roles in chromatin stability, remodelling or the accessibility of DNA (DNase and MNase are used to estimate DNA accessibility and nucleosome density respectively).

To investigate in more detail the localization of the top factors that correlate with the stationary distribution at the 100 kb scale, we identified regions of the genome that shows the highest stationary distribution values. Here we chose to focus on the top 10% of the stationary distribution values. We defined highly interacting regions as five or more consecutive 5 kb bins (the resolution of the Hi-C data that we used to calculate the stationary distribution) that falls within the top 10% stationary distribution values. We ended up with 787 highly interacting regions (HIRs) across the genome with an average length of 31.2 kb (See Materials and Methods, Figs S1, S3 and Supplementary information SI-6). We next calculated the average enrichment of the factors within the HIRs, within the flanking regions on each side of the HIRs, as well as within the two regions 100 kb upstream and downstream of the HIRs (HIRs-F1 and HIRs-F2, respectively, see Figs S3 and SI-5). Clearly, the most strongly correlating factors mentioned above that are all involved in DNA repair are clearly enriched specifically in genomic regions with the highest numbers of contacts (Figs S6, see S7A–C for localization of other factors). We conclude that enrichment of bound proteins involved in DNA damage repair correlates well with frequencies of DNA contacts, and is maximal in HIRs.

### Highly interacting regions tend to be fragile

**Figure 2:**
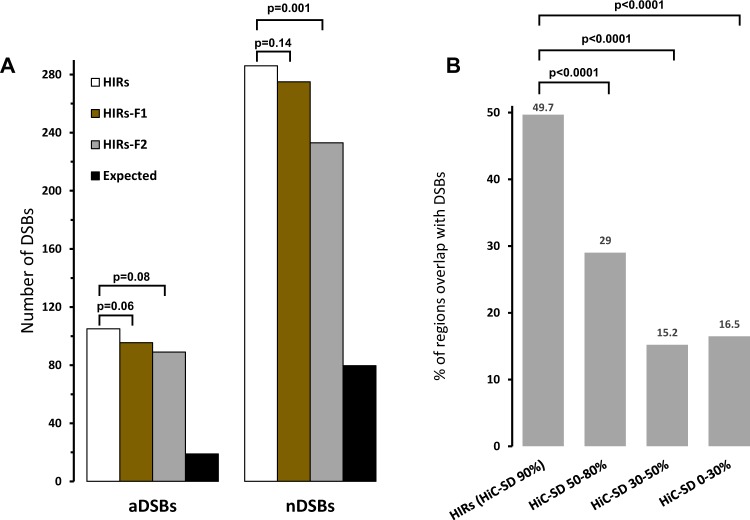
(**A**) Numbers of double stranded breaks (aDSBs and nDSBs denote aphidicolin- and neocarzinostatin-induced DSBs, respectively) within the HIRs, the two flanking regions and the expected genomic densities. (**B**) Percentages of regions based on four cut-offs of Hi-C stationary distribution (HiC-SD) overlapping with DSBs. HIRs have significantly more overlap with DSBs than the other three types of regions. Indicated p-values are based on student t-tests.

To explore the reasons for accumulation of DNA repair proteins in HIRs, we examined correlations between their distribution and reported sites of chemically-induced double strand breaks (DSBs). We mapped the HIRs to DSBs induced by aphidicolin (an inhibitor of DNA replication) and neocarzinostatin (which causes radiation-mimicking DNA damage) in HeLa cells, previously mapped using the BLESS method^30^. We selected 2343 and 6674 sites of the genome where aphidicolin and neocarzinostatin respectively have pronounced effects (with e-values ≤ 0.05). We found that HIRs overlap with about 4.5% of these DSBs within the genome: ~3- (aphidicolin) and ~5-fold (neocarzinostatin) more than expected if breaks were randomly distributed, based on HIRs’ 0.8% coverage of the human genome (Figs 2, SI-7). These findings indicate that HIRs are more fragile than other parts of the genome.

Moreover, our results show that genes overlapping the HIRs are about two times longer than average (52 vs 30 kb), in accordance with previous findings that long genes can induce instability^39,40^, and tend to be active. The average expression level in HIRs is 42 RPKM, more than twice the genomic average (19 RPKM), based on data presented in Fig. S8 and Supplementary Information SI-7. However, although the long overlapping genes tend to be active, we found that expression of short genes accounts for most of the expression in these regions. Additionally, HIRs harbour three times more than the average genomic density of ncRNAs (Fig. S8, Supplementary Information SI-7). Genes overlapping HIRs are involved in stress responses, immune responses through the interferon and cytokine pathways, cell-cell adhesion and regulation of apoptosis (Supplementary Information SI-8). The genes are also linked with cancer and various other disorders, *inter alia* autoimmune, inflammatory, and neurological diseases (Supplementary Information SI-8). These results strongly indicate that regions with high numbers of contacts tend to have longer and more active genes and to be more fragile than other parts of the genome.

### Highly interacting regions consists of different functional classes

**Figure 3:**
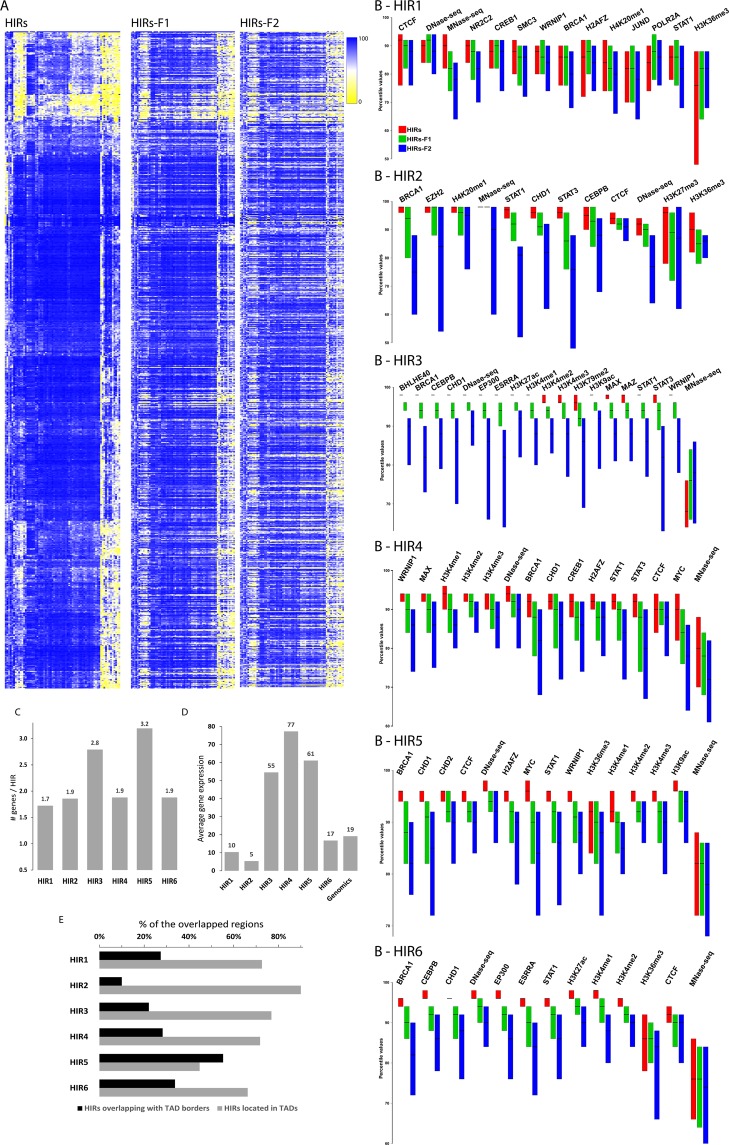
(**A**) Hierarchical clustering of HIRs (rows) and ENCODE factors (columns) based on Euclidian distances. HIR-F1 and HIR-F2 rows and columns are sorted as HIRs. (**B**) The boxplot shows enrichment of the most strongly enriched factors in the HIRs, relative to the two flanking regions for each class. The ChIP-seq values were normalized to the corresponding percentile values. (**C**) Average numbers of transcripts per HIR in each class. (**D**) Average gene expression levels (RPKM) in each HIR class (Fig. S11). (**E**) Percentages of the HIRs overlapping with TAD borders or completely localized within TADs.

As previously reviewed studies have shown that different functional types of genomic regions interact both locally and over large distances^14^, we investigated functional features of the identified HIRs. For this, we first created a heatmap showing associations between the HIRs (and two types of flanking regions) and enrichment of the ENCODE factors (Fig. 3A). The heatmap clearly shows substantial variation in the factors’ binding patterns, i.e., some bind strongly to some regions and rarely to other regions. Thus, the HIRs presumably represent different types of chromatin.

Next, we classified the HIRs by PCA and hierarchical clustering of enrichment values of 94 of the 177 ENCODE factors for the 787 HIRs, excluding factors with average enrichments in HIRs that were close to the genomic background or for which there were large amounts (>50%) of missing data (see *Material and Methods*). 83 of all 177 ENCODE datasets had a very low enrichment or had more than 50% missing data, and were therefore excluded. The six significant principal components (after centring and unit variance scaling of the variables) were subjected to Ward clustering and based on this clustering we defined six classes that we designated HIR1-HIR6 (Fig. S9). To investigate potential functions of the six classes we examined the most strongly enriched factors in these regions (Fig. 3B), and some other features such as numbers of genes and their expression levels (Fig. 3C,D and Supplementary Information SI-7). We also calculated how frequently HIRs of the same class and different classes interacted with each other, as described and illustrated in *Materials and Methods* and Fig. S10). Using these criteria, we divided the HIRs into three main groups, which are briefly described in the following sections.

#### Regions of repressed transcription and compact chromatin

Gene expression levels in HIR classes 1 and 2 are very low (Fig. 3D) and the genes tend to be very long (more than twice the genomic average, Fig. S11). In HIR1 regions, no factor is very strongly enriched, but CTCF is most strongly enriched, and they are more protected from MNase digestion (i.e., have high MNase-seq values, indicating high nucleosome occupancy) than surrounding chromatin (Fig. 3B). HIR2 regions also have high nucleosome occupancy, but they are also enriched with EZH2, a member of the Polycomb complex and H3K27me3, indicating that they are composed of Polycomb-repressed chromatin (Fig. 3B). Our observations suggest that Polycomb-repressed regions interact with other Polycomb-repressed regions in humans, as previously observed in *Drosophila*^41^ (Fig. S10).

#### Regions of high transcription

The regions classified as HIRs 3, 4 and 5 have very active transcription (Fig. 3D). Most of the expression in HIRs 4 and 5 is of short genes (Figs S11 and SI-7). Characteristics of HIR3 include strong (>4-fold) enrichment of ncRNAs (Fig. S11), RNA polymerase II and active histone marks, e.g. H3K4me1, H3K4me2 and H3K4me3, H3K36me3, H3K9ac and H3K27ac (Fig. 3B). They also seem to have open chromatin, according to DNase-seq data (Fig. 3B). A characteristic of HIR5 regions is a tendency to be located at TAD borders, while other HIRs are preferentially located within TADs (Fig. 3E). We conclude that HIRs 3, 4 and 5 are actively transcribed gene regions. HIR5 regions interact preferentially with regions of all three active classes (3, 4 and 5), while HIR4 regions only seem to interact with HIR5 regions (Fig. S10). While these preferential interactions are intriguing, we speculate that these three classes could represent so-called transcriptional factories^42^.

#### Regions of repressed transcription and open chromatin

Gene expression levels within HIR6 regions are very low (Fig. 3D), the genes in them tend to be very long (Fig. S11), and active histone marks are depleted. However, they seem to consist of open and accessible euchromatic regions of the genome, as indicated by high DNase-seq values and low MNase-seq values (Fig. 3B). Taken together, these findings suggest that genes in HIR6 regions are repressed, but not associated with compact chromatin.

### Regions with long-range interactions are enriched in predicted enhancers

**Figure 4:**
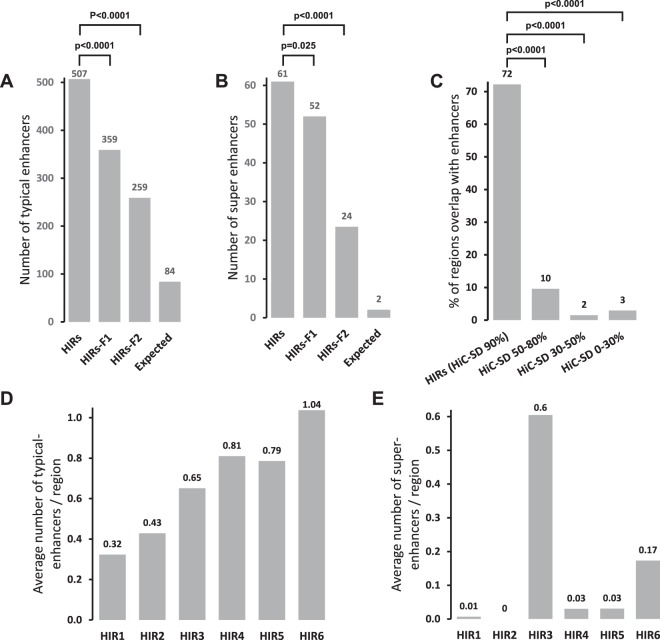
Numbers of (**A**) typical-enhancers and (**B**) super-enhancers overlapping HIRs, the two flanking regions and expected densities. (**C**) Percentages of regions, based on four cut-offs of Hi-C stationary distribution (HiC-SD) overlapping typical- and super-enhancers (combined). (**D**) Average numbers of typical-enhancers and (**E**) super-enhancers overlapping each HIR class. (**F**) Average numbers of ncRNAs overlapping each HIR class. P-values derived from Student t-tests are indicated.

It was recently reported that highly interacting regions at the local scale (<200 kb), called FIRE regions, are strongly enriched with predicted enhancers and super-enhancers^29^. Therefore, we investigated correlations between localizations of the same predicted enhancers from^28^ and the HIR regions we identified, based on all interactions across each chromosome (including long-range interactions). The results show that HIRs overlap with 8% of the typical enhancers and 25% of the super-enhancers, corresponding to 6- and 29-fold higher than average densities in the genome, respectively (Fig. 4A,B). Both types of enhancers are also enriched in the flanking regions, and HIRs are clearly very strongly enriched in enhancers (Fig. 4C).

Within classes we observed that HIRs 3, 4, 5 and 6 are most strongly enriched in typical enhancers (Fig. 4D) and that HIRs 3 and 6 are also strongly enriched in super-enhancers (Fig. 4E). We note that HIRs 3 and 6 also interact strongly within and between themselves (Fig. S10), indicating that enhancers and especially super-enhancers are involved in long-range interactions in the genome.

## Discussion

In this study we computationally defined regions of the human genome that have high numbers of intra-chromosomal contacts. Unlike TADs, these regions are not solely in contact with chromosomal regions that are in close proximity in linear space. Rather, they represent higher order three-dimensional structures that bring together distant regions located on different parts of chromosomes. Regions on different chromosomes also interact quite extensively^24^, but due to limitations in the Hi-C data we could not investigate their relative frequencies^43^. We found that highly interacting regions (HIRs) of the genome have higher levels of bound DNA damage repair factors than other genomic regions, and tend to be more fragile. Moreover, we observed that HIRs are enriched in enhancers and super-enhancers. These observations are consistent with recent demonstrations that highly interacting loop anchors are fragile and enriched in double strand breaks^44^, and chromosomal regions with high levels of local chromatin interactions are enriched in super-enhancers^29^.

Microscopic observations have revealed that genomic regions undergoing DNA repair may be moved up to 2 μM from their normal nuclear territory^14,22,45^. Recent studies have also shown that regions undergoing repair can be brought into contact and cluster^23^. These findings, together with results presented here, indicate that the most highly interacting genomic regions may represent repair factories. However, it seems unlikely that all HIR regions are being actively repaired, and our results indicate that they are brought together for other functional reasons.

Although debated, previous studies have indicated that regions with similar function can interact over long distances, such as the clustering of transcriptionally active loci^46^ (sometimes referred to as transcription factories) and clustering of Polycomb repressed regions (so called Polycomb bodies) in fruit flies^14^. We suggest that the HIR3, 4 and 5 classes we identify are clustered transcriptionally active sites of the human genome, whereas the HIR2 class could represent clusters of Polycomb repressed chromatin.

We also found that regions of inactive compact chromatin can have high numbers of contacts (HIRs 1 and 2). The HIR1 regions also seem to lack strong binding of any ENCODE factors, but they have high nucleosome density according to MNase-seq data. Large genomic regions lacking enrichment of virtually all mapped factors have also been observed in fruit flies^47^. These regions have been termed null or black chromatin, and are transcriptionally inactive. The compaction of the chromatin likely contributes to the high numbers of contacts observed in HIR1 regions.

For the first time we here report the interaction of regions of transcriptionally inactive but open chromatin. These (HIR6) regions are enriched in H3K27ac, H3K4me1 and EP300. They are also strongly enriched in previously defined predicted enhancers and (especially) super-enhancers^28^. This is consistent with previous reports of high numbers of local Hi-C contacts in enhancer-rich regions^29^. Active enhancers are expected to generate several contacts with neighbouring loci. Interestingly, we found that HIR6 regions interact strongly with other HIR6 regions and HIR3 regions (the two classes with the highest numbers of predicted super-enhancers). We therefore suggest that super-enhancers are involved in long-range interactions in the genome. Super-enhancers have previously been shown to contain HOT-regions, which have very high densities of bound transcription factors^48^. It has also been noted that several transcription factors localize in HOT regions, despite absence of their target motifs. Although many transcription factors are probably recruited in such regions through physical interactions with other transcription factors we speculate that they could also be cross-linked there through three-dimensional interactions with other HOT regions.

Although the HIRs we identified seem to interact for functional reasons, it is intriguing that they are enriched in DNA repair factors. Bringing functionally related regions of the genome into physical proximity presumably has selective advantages as it should help regulatory machinery to locate relevant loci rapidly. However, bringing many loci from separate genomic origins together raises risks of detrimental chromosomal rearrangements through improper repair of DNA damage. Accordingly, there seems to be a correlation between regions forming long-range interactions and chromosomal rearrangements^24^, and the relationship between three-dimensional organization and DNA repair has been previously discussed^49^. We speculate that DNA damage repair factors are enriched in HIRs because of the selective advantage it provides through enabling rapid repair of DNA damage and thus reduction of risks of catastrophic genomic rearrangements.

## Supplementary information

Supplementary information SI-1

Supplementary information SI-2

Supplementary information SI-3

Supplementary information SI-4

Supplementary information SI-5

Supplementary information SI-6

Supplementary information SI-7

Supplementary information SI-8

Supplementary information SI-9

Supplementary information SI-10

## Data Availability

The stationary distribution method is implemented in the gcMapExplorer package (https://github.com/rjdkmr/gcMapExplorer).
