# Supplementary material for: Highly interacting regions of the human genome are enriched with enhancers and bound by DNA repair proteins
Source: Sci Rep. 2019 Mar 14;9:4577. doi: 10.1038/s41598-019-40770-9 (PMC6418152; doi:10.1038/s41598-019-40770-9)
Supplement: Supplementary file 1 — Supplementary information SI-1 [file 41598_2019_40770_MOESM1_ESM.pdf]

Highly interacting regions of the human genome are enriched with enhancers and bound by DNA repair proteins

Haitham Sobhy, Rajendra Kumar, Jacob Lewerentz, Ludvig Lizana and Per Stenberg

Supplementary information SI-1. This document contains table and figure legends.

Supplementary information SI-2. HiC stationary distribution (HiC-SD) values (sgr format).

Supplementary information SI-3. The ENCODE ChIP-seq datasets used in the current study. The official gene name, proteins' uniprot ID and ChIP-seq ENCODE ID are listed.

Supplementary information SI-4. The top correlated ChIP-seq data with HiC-SD at 100kb resolution. WRNIP1 and NR2C2 have high R in chromosomes 2, 6, 7, 8, 9, and 10; whereas, MYC, BRCA1 or STAT1 have high R in chromosomes 1, 11, 21, 22, and X.

Supplementary information SI-5. HiC-SD values at different cut-offs, Hi-C SD peaks with values larger than 90% of the SD, values between 50-80% of the SD, 30-50% and the lowest values from 0 to 30% of Hi-C SD.

Supplementary information SI-6. A bed format file contains the coordinates of the highly interacting regions (HIRs). The different classes are coloured according to the following colour code: HIR1: black; HIR2: fuchsia; HIR3: yellow; HIR4: blue; HIR5: red; and HIR6: green.

Supplementary information SI-7. The data used to construct the figures. The file contains the HIR regions and the overlapping genes, enhancers, expression level, etc.

Supplementary information SI-8. Gene ontology of the genes overlapping the HIRs as shown by (*p-value* <0.05), PANTHER (http://pantherdb.org/, and http://geneontology.org/), DAVID (https://david.ncifcrf.gov/), and GREAT tool (http://bejerano.stanford.edu/great/).

Supplementary information SI-9. Percentile values of ChIP-seq data within the six classes and the p-values.

Supplementary information SI-10. HIR localization with respect to TAD borders.

Supplementary information SI-1.

Supplementary Tables

Chr #	Start	End	Length
chr11	60590000	60615000	25000
chr11	62355000	62395000	40000
chr11	64680000	64705000	25000
chr11	64785000	64810000	25000
chr11	64830000	64870000	40000
chr11	65115000	65155000	40000
chr11	65625000	65650000	25000
chr11	66840000	66865000	25000
chr11	67410000	67435000	25000
chr11	68865000	68890000	25000
chr11	72120000	72145000	25000
chr11	72390000	72415000	25000
chr11	72450000	72475000	25000
chr11	72480000	72520000	40000
chr11	72885000	72910000	25000
chr11	72930000	72955000	25000
chr11	72990000	73015000	25000
chr11	73080000	73120000	40000
chr11	73335000	73375000	40000
chr11	73725000	73750000	25000
chr11	75030000	75055000	25000
chr11	75150000	75220000	70000
chr11	75240000	75265000	25000
chr11	75840000	75865000	25000
chr11	75885000	75910000	25000
chr11	75930000	75955000	25000
chr11	76365000	76405000	40000
chr11	76875000	76900000	25000
chr11	77880000	77905000	25000

Table S1. The highly interacting regions (HIRs) with top 10% of Hi-C stationary distribution (5kb resolution) in chromosome 11, and visualized in Fig. 1A. The neighbouring HIRs regions were merged.

Supplementary Figures

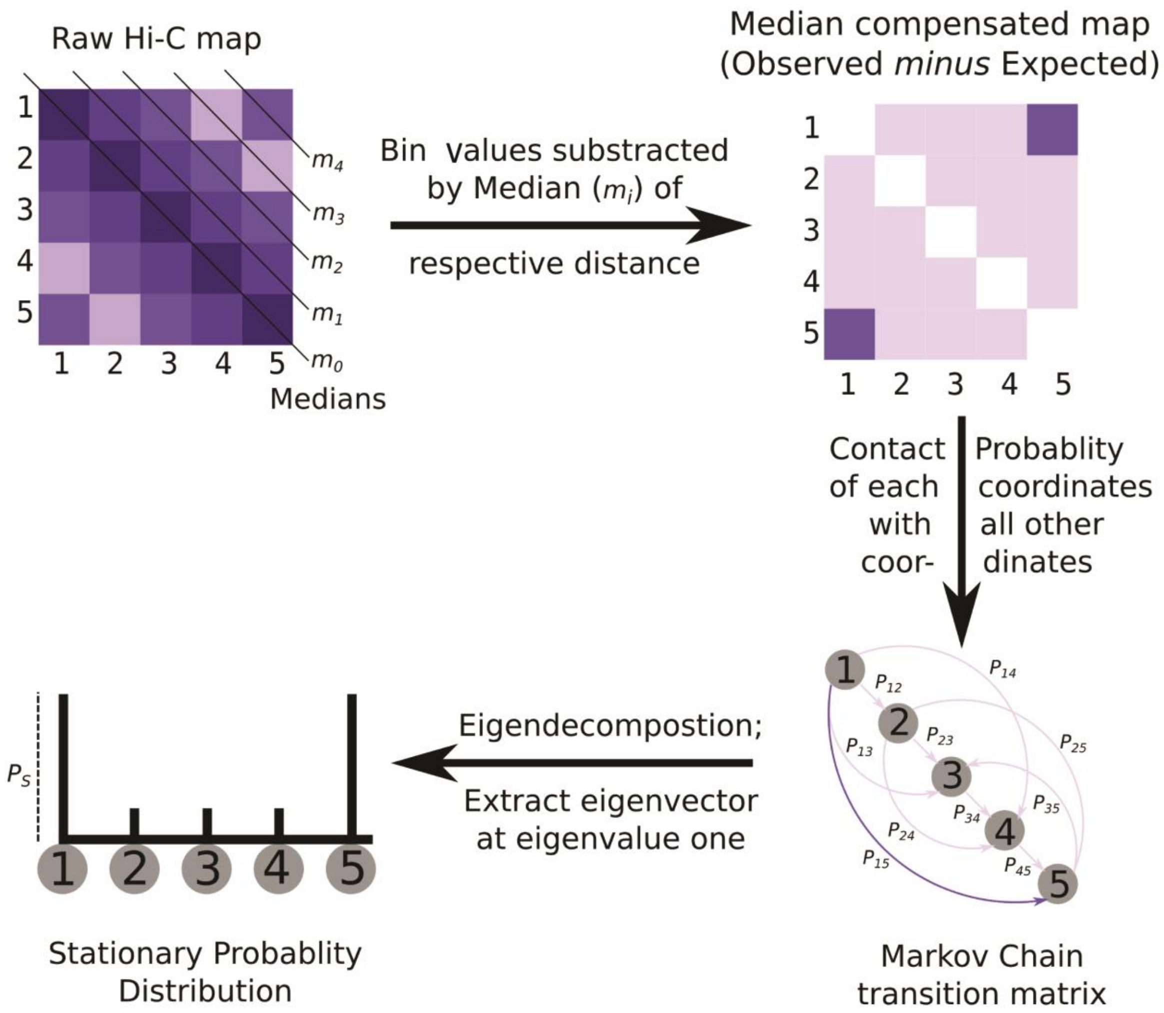

Fig. S1. A representative illustration of the method used to calculate stationary distribution

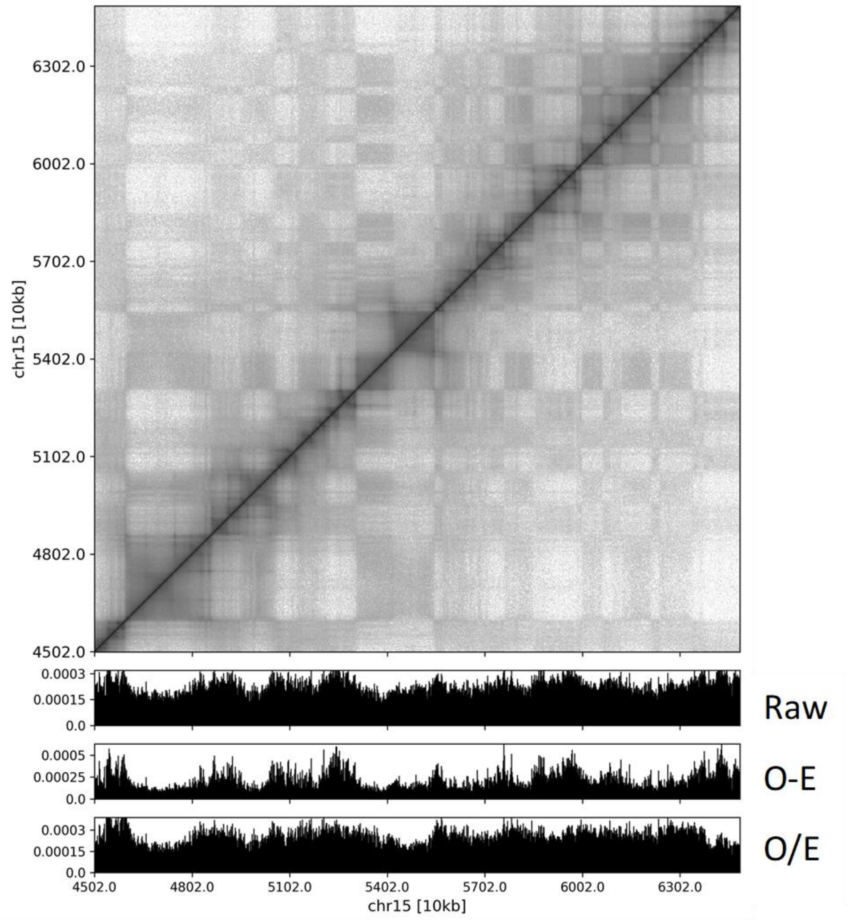

Fig. S2. The profile of raw, O/E and O-E stationary distribution (SD).

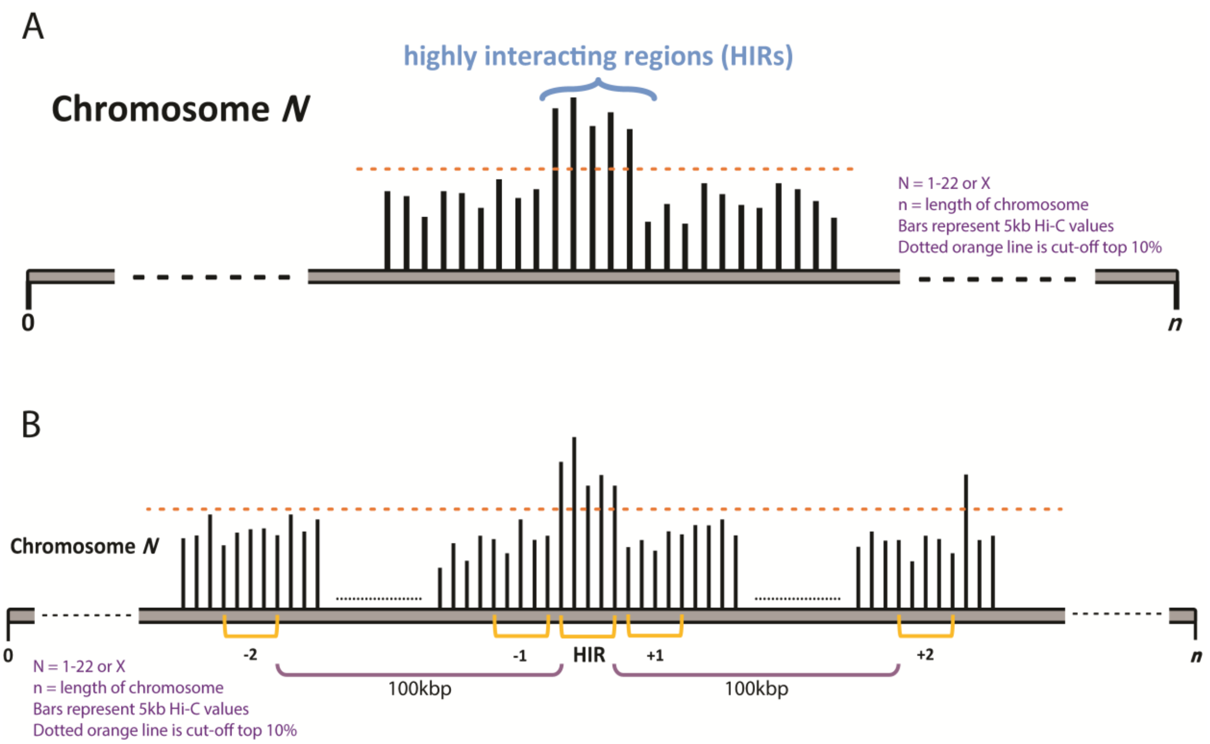

Fig. S3. The method of identifying highly interacting regions (HIRs). (A) Definition of the HIRs as five consecutive 5kb Hi-C SD peaks that are top 10%. (B) Four flanking regions were identified, two before and two after the HIRs, and the flanking regions.

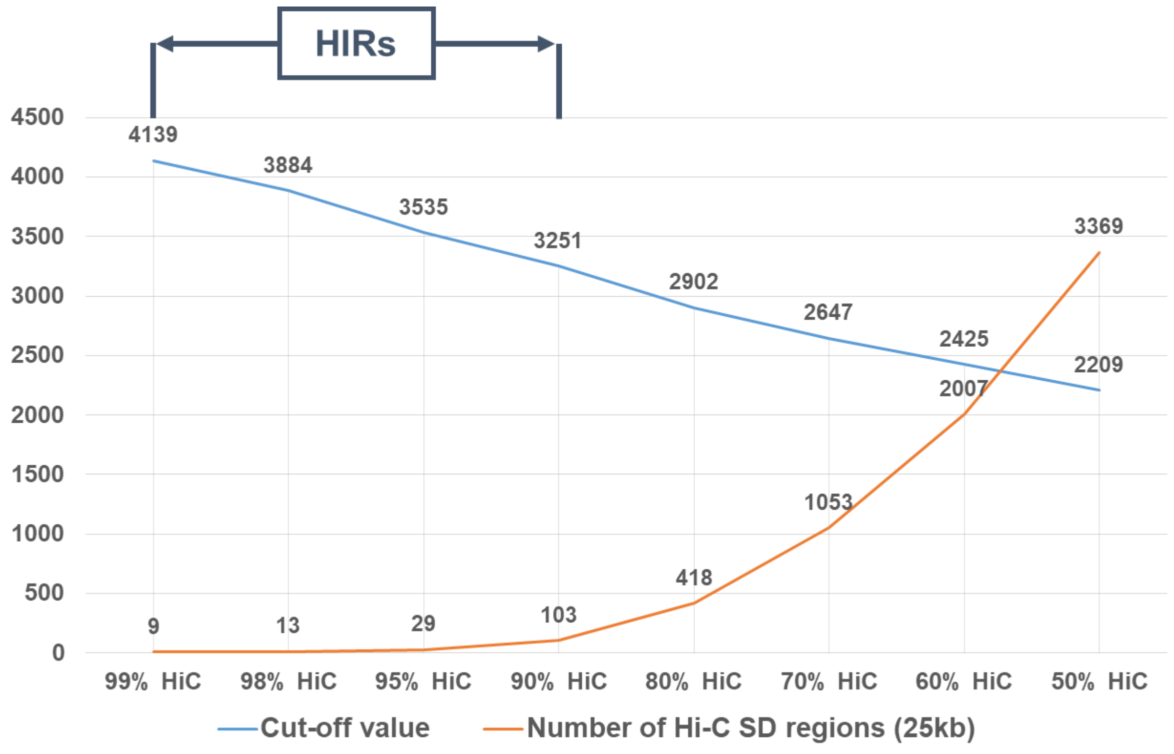

Fig. S4. The number of Hi-C SD regions in chromosome 1 with respect to different percentile cut-offs values. The length of the regions is 25kb, i.e. five consecutive 5kb Hi-C SD peaks, as shown in Fig. S3. The highly interacting regions (HIRs) are top 10% regions. The overlapped regions are merged for downstream analysis.

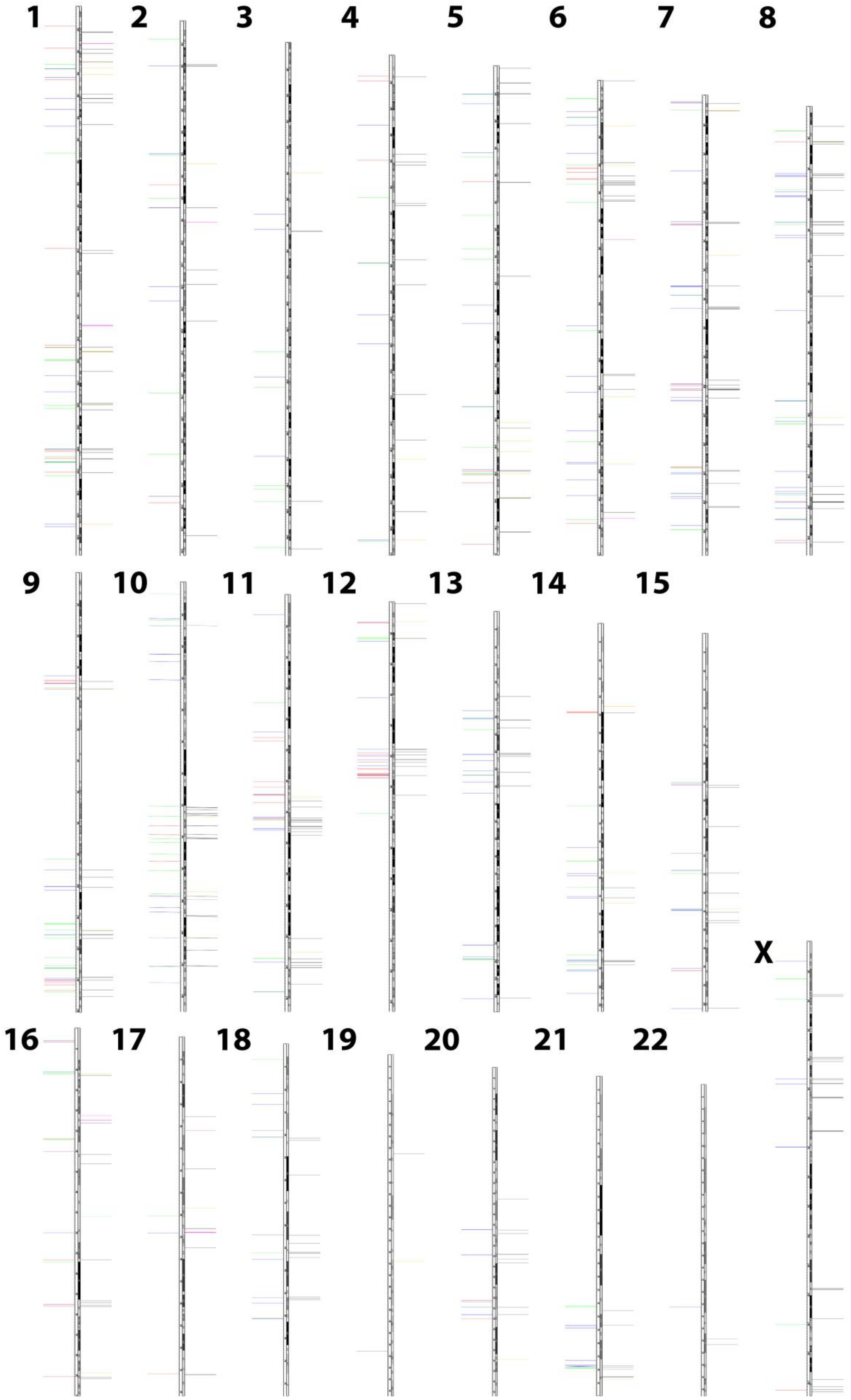
1
2
3
4
5
6
7
8
9
10
11
12
13
14
15
X
16
17
18
19
20
21
22

Fig. S5. The location of the HIRs on the chromosomes are shown. The six classes are coloured as the following: HIR1: black; HIR2: fuchsia; HIR3: yellow; HIR4: blue; HIR5: red; and HIR6: green. The chromosome numbers are indicated above.

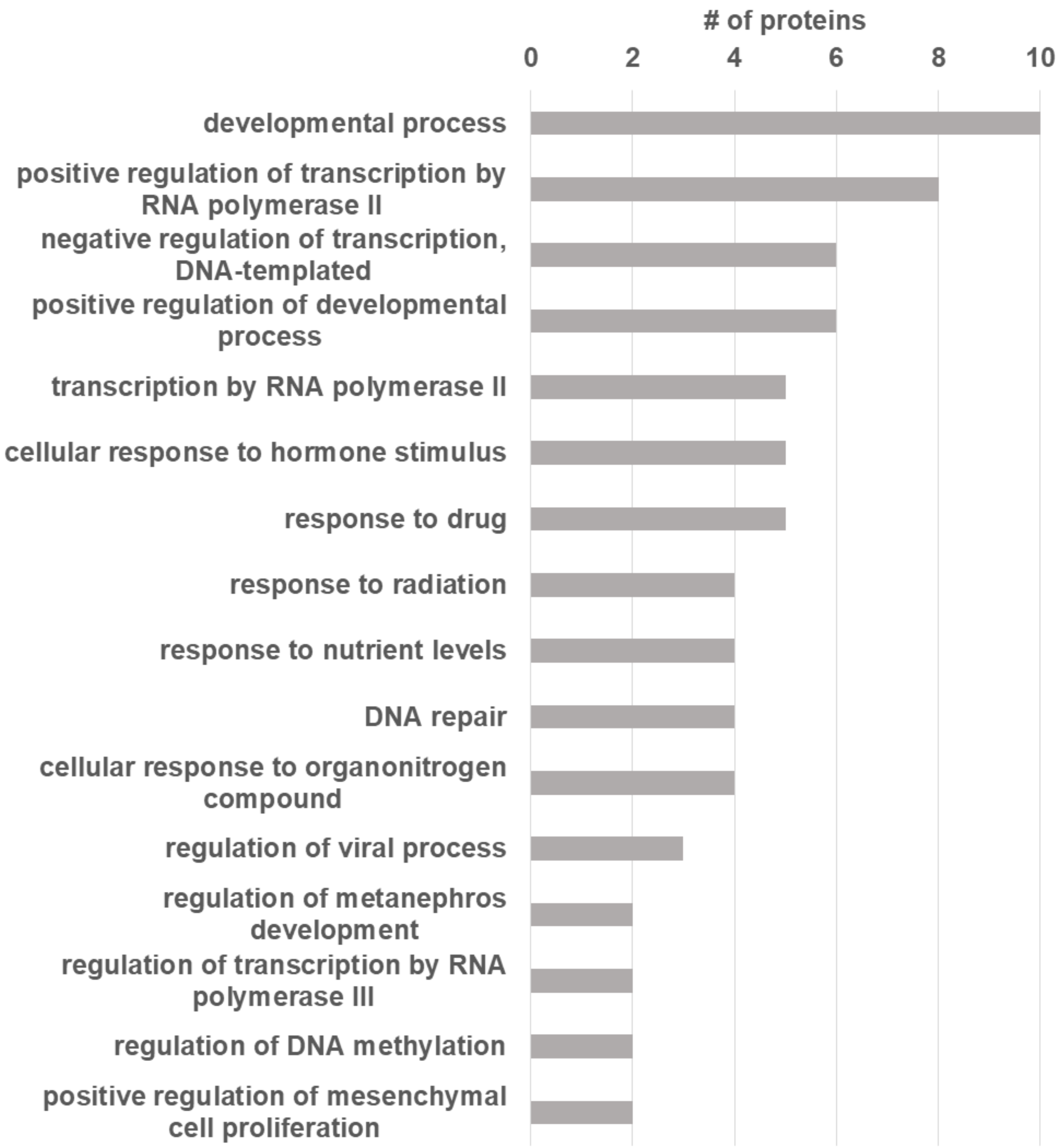

Fig. S6. The GO terms for top correlated ChIP-seq data (14 proteins). The analysis was performed by PANTHER overrepresentation database (http://pantherdb.org/, which is belong to Gene Ontology Consortium, http://www.geneontology.org/) using the default parameters (GO biological process, Fisher's exact test and calculate false discovery rate <0.05). The GO terms with p-values <0.05 are shown. .

7A

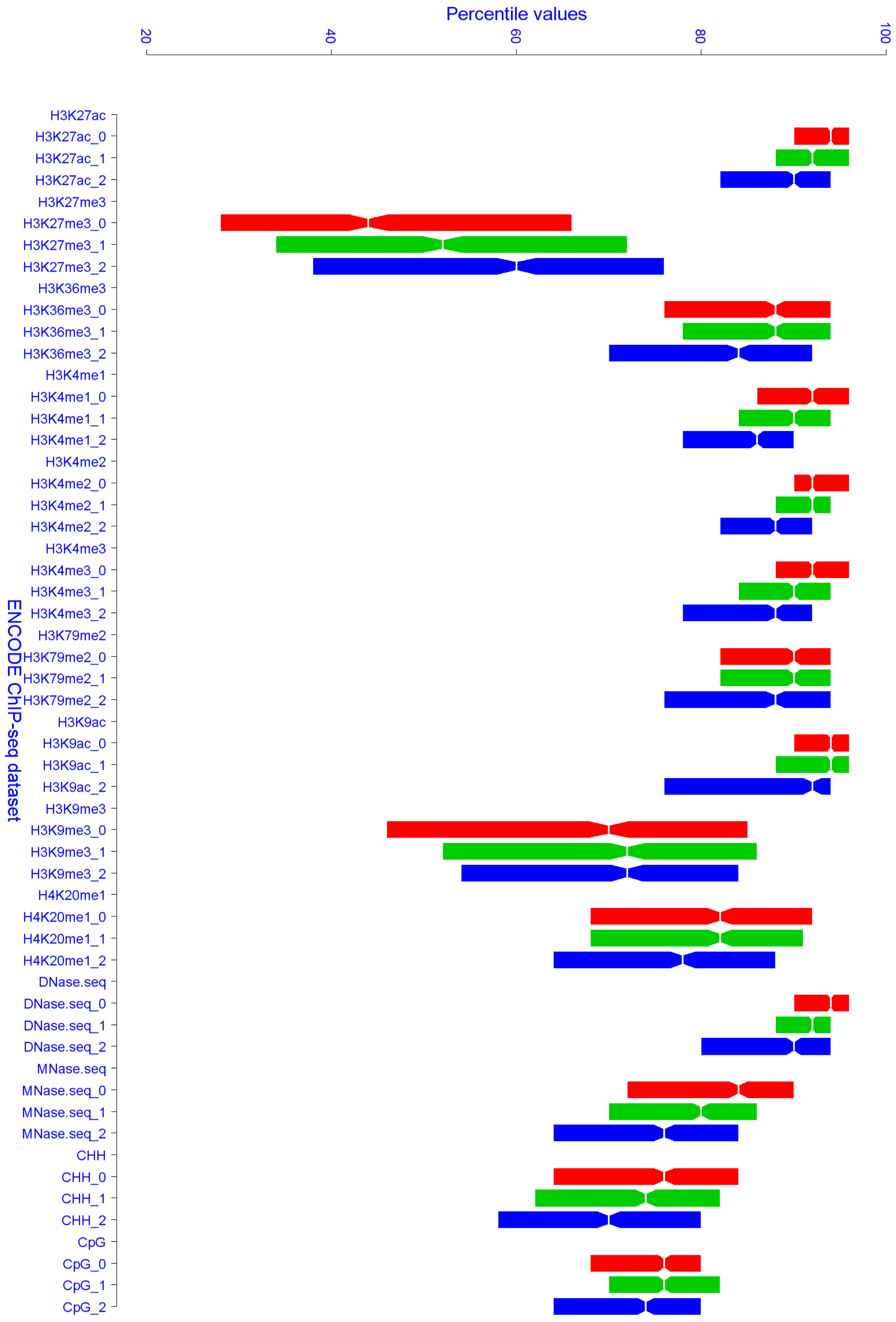
Percentile values
20
40
60
80
100
ENCODE ChIP-seq dataset
H3K27ac
H3K27ac_0
H3K27ac_1
H3K27ac_2
H3K27me3
H3K27me3_0
H3K27me3_1
H3K27me3_2
H3K36me3
H3K36me3_0
H3K36me3_1
H3K36me3_2
H3K4me1
H3K4me1_0
H3K4me1_1
H3K4me1_2
H3K4me2
H3K4me2_0
H3K4me2_1
H3K4me2_2
H3K4me3
H3K4me3_0
H3K4me3_1
H3K4me3_2
H3K79me2
H3K79me2_0
H3K79me2_1
H3K79me2_2
H3K9ac
H3K9ac_0
H3K9ac_1
H3K9ac_2
H3K9me3
H3K9me3_0
H3K9me3_1
H3K9me3_2
H4K20me1
H4K20me1_0
H4K20me1_1
H4K20me1_2
DNase.seq
DNase.seq_0
DNase.seq_1
DNase.seq_2
MNase.seq
MNase.seq_0
MNase.seq_1
MNase.seq_2
CHH
CHH_0
CHH_1
CHH_2
CpG
CpG_0
CpG_1
CpG_2

7B

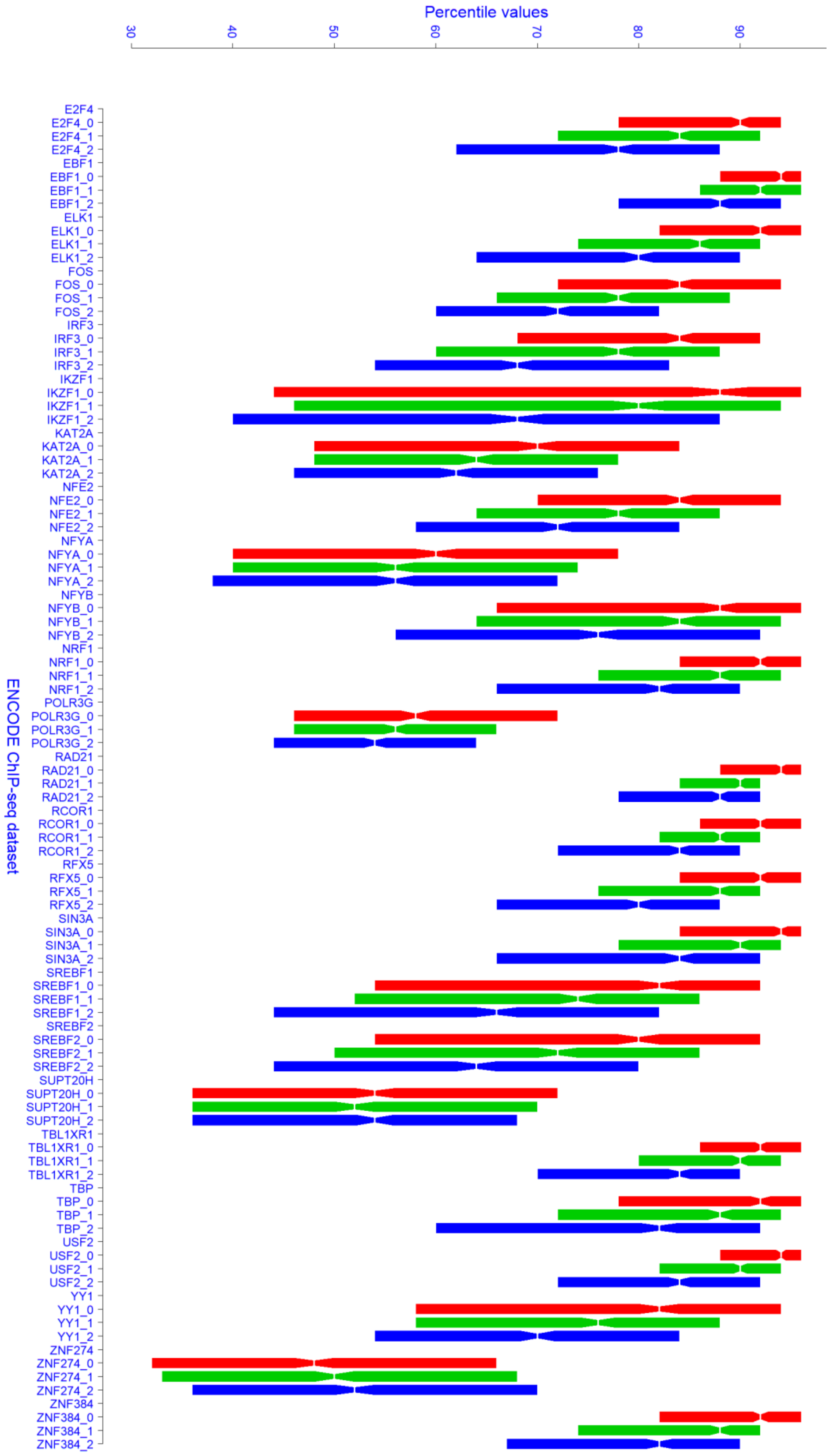

Percentile values
30
40
50
60
70
80
90
ENCODE ChIP-seq dataset
E2F4
E2F4_0
E2F4_1
E2F4_2
EBF1
EBF1_0
EBF1_1
EBF1_2
ELK1
ELK1_0
ELK1_1
ELK1_2
FOS
FOS_0
FOS_1
FOS_2
IRF3
IRF3_0
IRF3_1
IRF3_2
IKZF1
IKZF1_0
IKZF1_1
IKZF1_2
KAT2A
KAT2A_0
KAT2A_1
KAT2A_2
NFE2
NFE2_0
NFE2_1
NFE2_2
NFYA
NFYA_0
NFYA_1
NFYA_2
NFYB
NFYB_0
NFYB_1
NFYB_2
NRF1
NRF1_0
NRF1_1
NRF1_2
POLR3G
POLR3G_0
POLR3G_1
POLR3G_2
RAD21
RAD21_0
RAD21_1
RAD21_2
RCOR1
RCOR1_0
RCOR1_1
RCOR1_2
RFX5
RFX5_0
RFX5_1
RFX5_2
SIN3A
SIN3A_0
SIN3A_1
SIN3A_2
SREBF1
SREBF1_0
SREBF1_1
SREBF1_2
SREBF2
SREBF2_0
SREBF2_1
SREBF2_2
SUPT20H
SUPT20H_0
SUPT20H_1
SUPT20H_2
TBL1XR1
TBL1XR1_0
TBL1XR1_1
TBL1XR1_2
TBP
TBP_0
TBP_1
TBP_2
USF2
USF2_0
USF2_1
USF2_2
YY1
YY1_0
YY1_1
YY1_2
ZNF274
ZNF274_0
ZNF274_1
ZNF274_2
ZNF384
ZNF384_0
ZNF384_1
ZNF384_2

7C

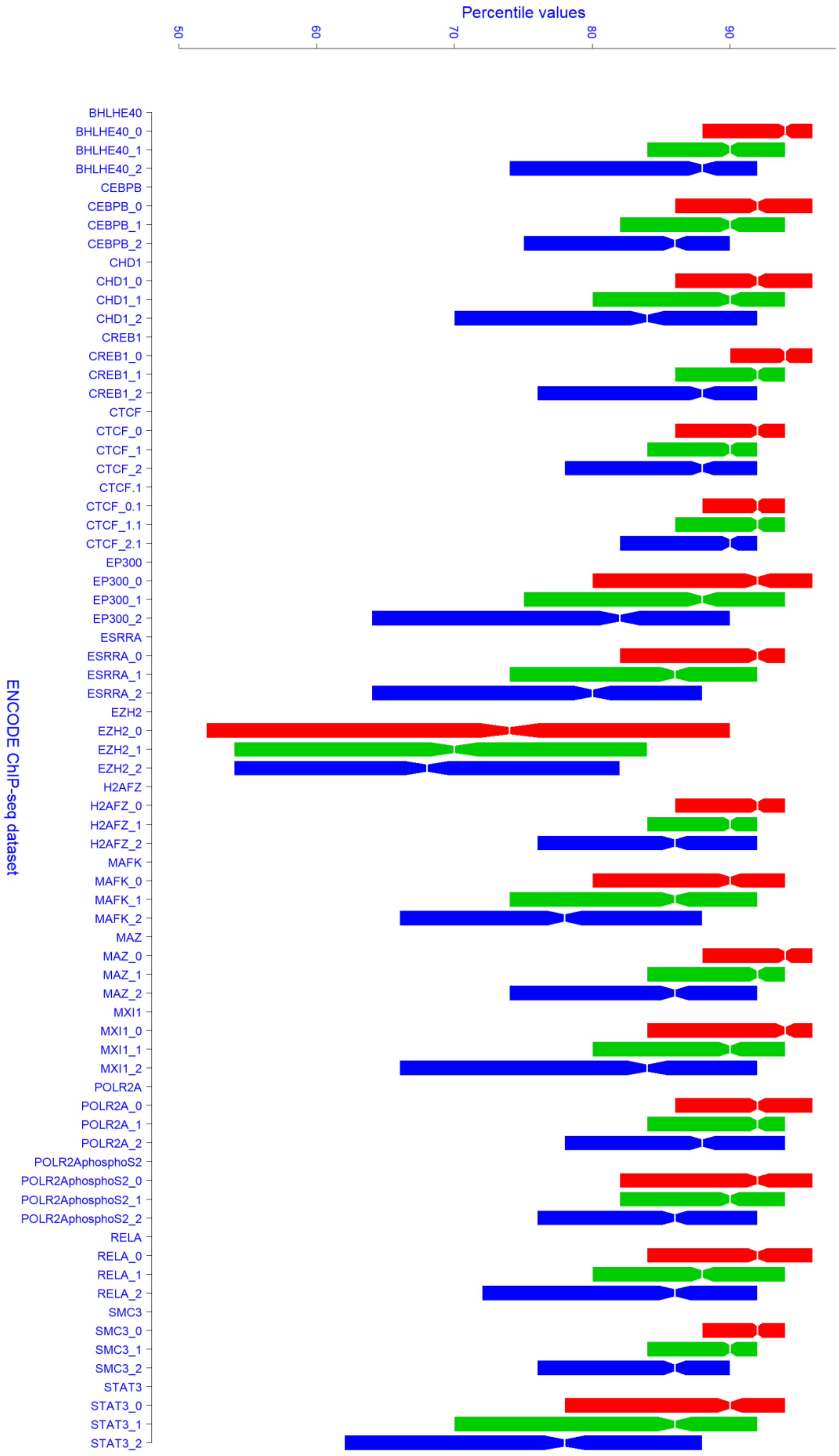
Percentile values
50
60
70
80
90
ENCODE ChIP-seq dataset
BHLHE40
BHLHE40_0
BHLHE40_1
BHLHE40_2
CEBPB
CEBPB_0
CEBPB_1
CEBPB_2
CHD1
CHD1_0
CHD1_1
CHD1_2
CREB1
CREB1_0
CREB1_1
CREB1_2
CTCF
CTCF_0
CTCF_1
CTCF_2
CTCF.1
CTCF_0.1
CTCF_1.1
CTCF_2.1
EP300
EP300_0
EP300_1
EP300_2
ESRRA
ESRRA_0
ESRRA_1
ESRRA_2
EZH2
EZH2_0
EZH2_1
EZH2_2
H2AFZ
H2AFZ_0
H2AFZ_1
H2AFZ_2
MAFK
MAFK_0
MAFK_1
MAFK_2
MAZ
MAZ_0
MAZ_1
MAZ_2
MXI1
MXI1_0
MXI1_1
MXI1_2
POLR2A
POLR2A_0
POLR2A_1
POLR2A_2
POLR2AphosphoS2
POLR2AphosphoS2_0
POLR2AphosphoS2_1
POLR2AphosphoS2_2
RELA
RELA_0
RELA_1
RELA_2
SMC3
SMC3_0
SMC3_1
SMC3_2
STAT3
STAT3_0
STAT3_1
STAT3_2

Fig. S7. Boxplots of ENCODE-factor ChIP-seq enrichment in the highly interacting regions (HIRs) and the flanking regions. The values are normalized to the corresponding percentile value and the first, second and third quartiles are shown. A, B and C are different type of factors. The 0 after the factor name denotes the HIR regions, while 1 and 2 denote the average of the first and second flanking regions, respectively. Multiple datasets were used.

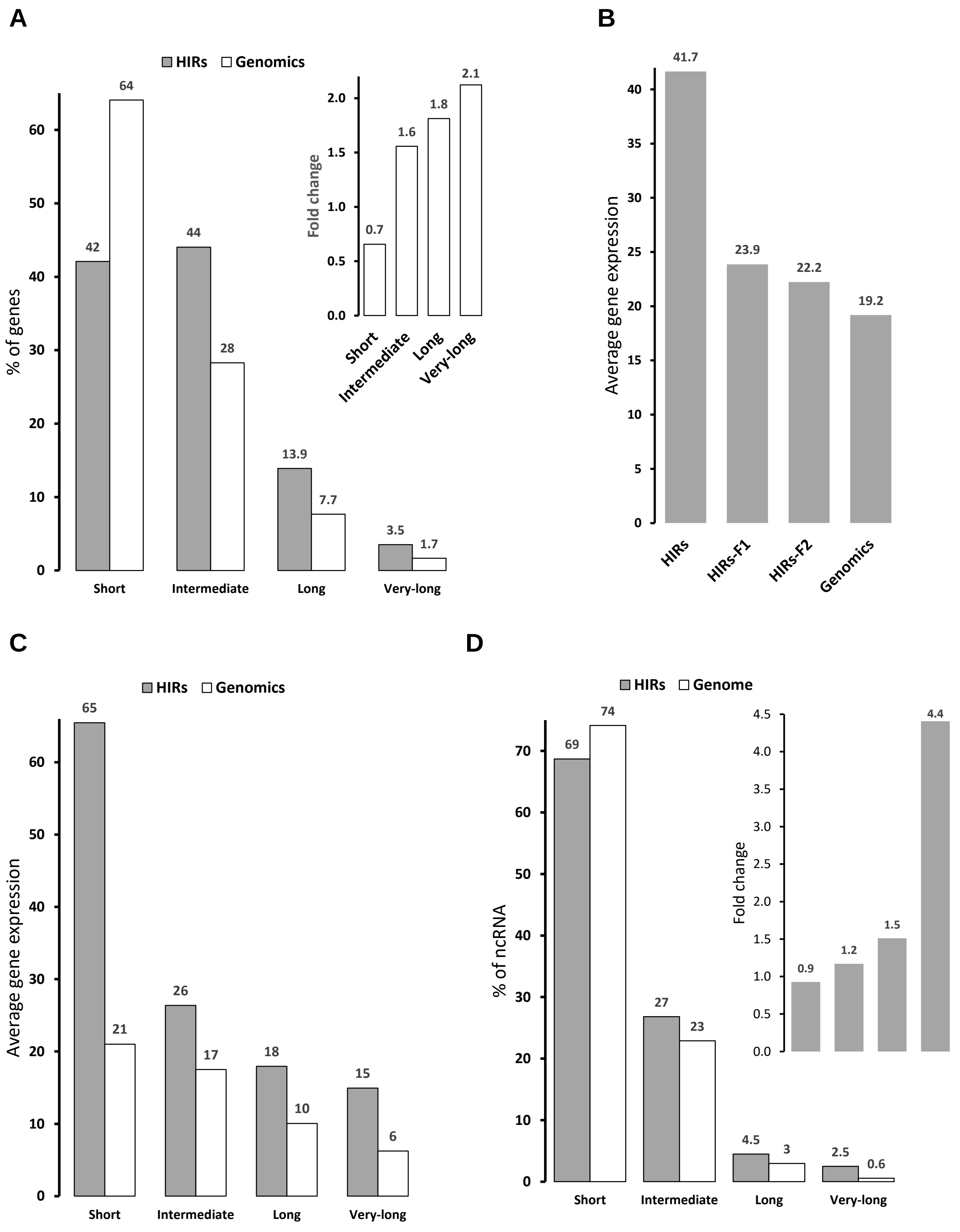
A
HIRs Genomics
% of genes
42
64
44
28
13.9
7.7
3.5
1.7
Short
Intermediate
Long
Very-long
Fold change
0.7
1.6
1.8
2.1
B
Average gene expression
41.7
23.9
22.2
19.2
HIRs
HIRs-F1
HIRs-F2
Genomics
C
HIRs Genomics
Average gene expression
65
21
26
17
18
10
15
6
D
HIRs Genome
% of ncRNA
69
74
27
23
4.5
3
2.5
0.6
Fold change
0.9
1.2
1.5
4.4

Fig. S8. (A) The percent of the genes overlapping with HIRs compared to the genomic distribution with respect to gene length. We divided the genes into four classes: The short genes are less than 10kb length, intermediate genes are 10-100kb; long are above 100kb and very-long genes are above 300kb. Note that very-long genes are a subset of the long genes. The small white bar graph shows the fold change of the genes overlapping HIRs relative to the genomic distribution. (B) The average gene expression (number of read) per transcript in HIRs, the two flanking regions and the genomics average. (C) The average transcription of each gene category within HIRs and the genome average. (D) The percent of ncRNAs overlapping with HIRs compared to the genomic distribution with respect to length, as well as the fold change relative to the genomic distribution.

A.

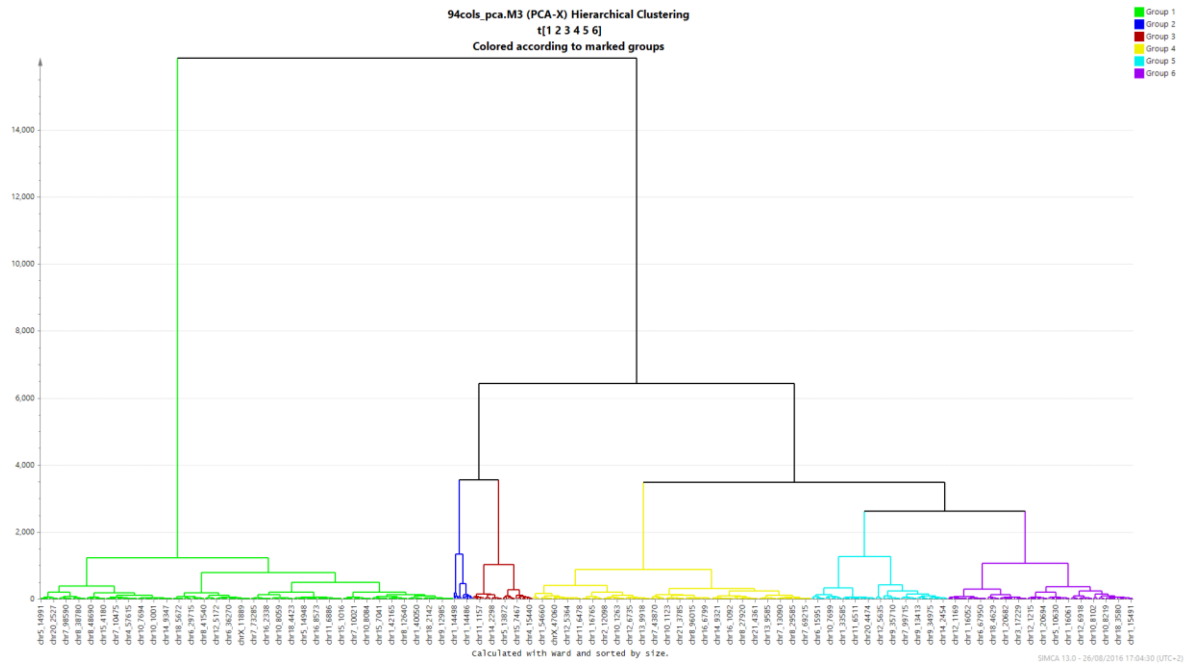

B.

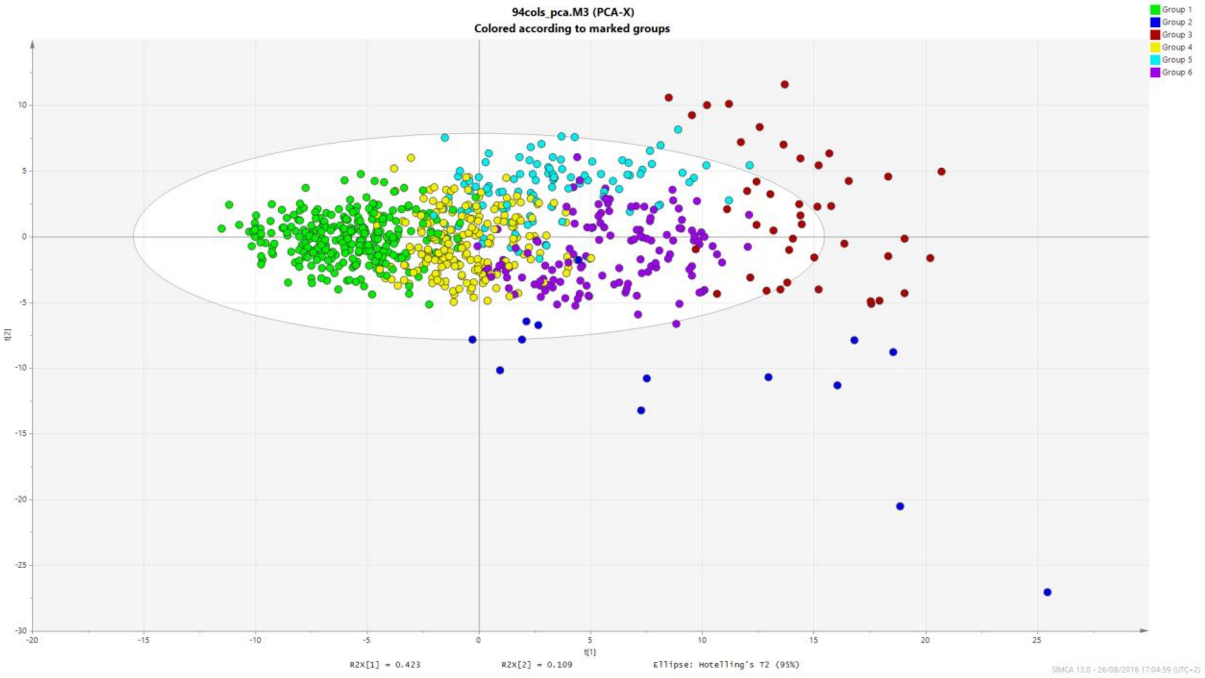

Fig. S9. The PCA cluster analysis using six components were HIRs are classified into six classes. The dendrogram shows the six classes (A) and the scatter plot (B) shows the first two components and the different classes.

HCRs1

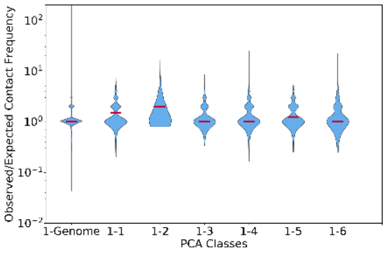

HCRs2

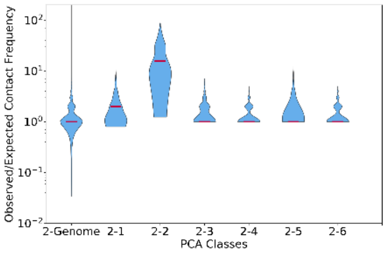

HCRs3

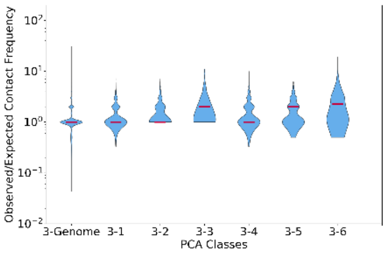

HCRs4

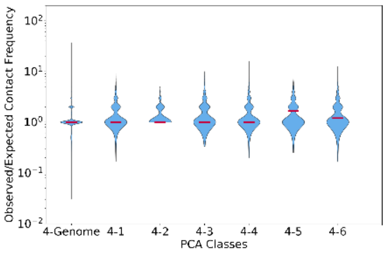

HCRs5

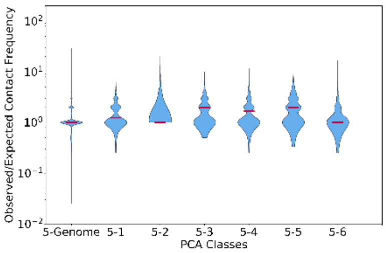

HCRs6

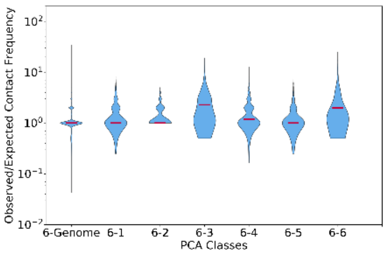

Fig. S10. The violin plot shows the distribution of the contact frequencies between HIR regions in each class with the genome, its own class regions and regions belong to other classes. The red bar indicate median values.

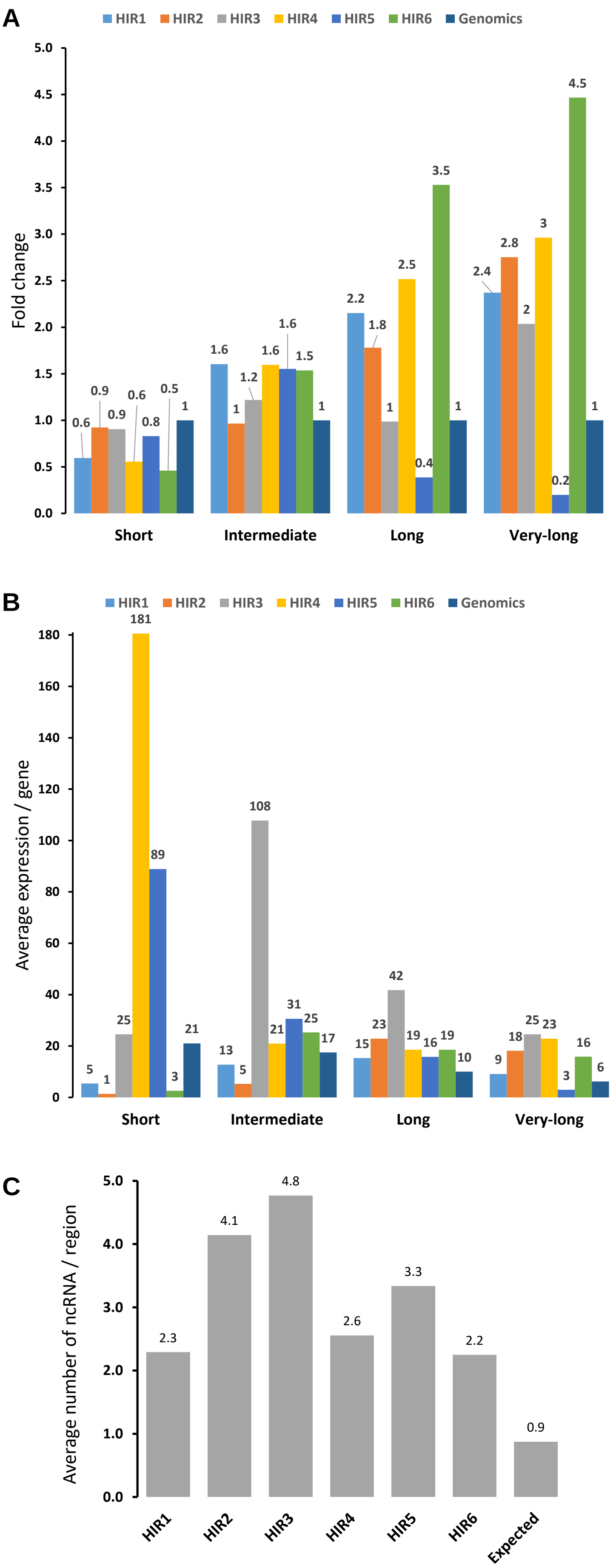
A
HIR1 HIR2 HIR3 HIR4 HIR5 HIR6 Genomics
Fold change
Short
Intermediate
Long
Very-long
B
HIR1 HIR2 HIR3 HIR4 HIR5 HIR6 Genomics
Average expression / gene
Short
Intermediate
Long
Very-long
C
Average number of ncRNA / region
HIR1
HIR2
HIR3
HIR4
HIR5
HIR6
Expected

Fig. S11. (A) The fold change of the genes overlapping the six HIR classes relative to the genomic distribution. (B) The average expression of the gene categories within each HIR class as well as the genome average. (C) Average number of ncRNA per region within classes and the expected genomics value.
